# Spontaneous necrotizing granuloma of the cerebellum: a case report

**DOI:** 10.1186/s12883-020-01814-0

**Published:** 2020-06-05

**Authors:** Kerilyn N. Godbe, Brian F. Saway, Evin L. Guilliams, John J. Entwistle, Robert W. Jarrett

**Affiliations:** 1 Virginia Tech Carilion School of Medicine, 2 Riverside Circle, Roanoke, VA 24016 USA; 2 Department of Neuroscience, Medical University of South Carolina, 171 Ashley Ave, Charleston, SC 29425 USA; 3 Carilion Clinic Division of Neurosurgery, 1906 Belleview Ave. SE, Roanoke, VA 24014 USA; 4 Department of Basic Science Education, Virginia Tech Carilion School of Medicine, 1 Riverside Circle, Suite 105, Roanoke, VA 24016 USA

**Keywords:** CNS, Necrotizing granuloma, Spontaneous, ANCA, Cerebellum

## Abstract

**Background:**

Intracranial necrotizing granulomatous space-occupying lesions are sparsely reported in literature. Variability in presenting symptomatology and radiographic features makes diagnostic work-up difficult.

**Case presentation:**

This report presents the case of a 77-year-old female with sinusitis and fatigue who underwent an MRI revealing a posterior fossa lesion compressing the fourth ventricle. Subsequent contrast CT of the chest, abdomen, and pelvis was negative for primary malignancy. Histopathologic examination of the lesion following biopsy showed it to be a necrotizing granuloma in an antineutrophil cytoplasmic antibody (ANCA) negative patient. The most likely diagnosis was determined to be spontaneous necrotizing granuloma, a rare entity with only one previous report noted.

**Conclusions:**

Spontaneous necrotizing granuloma of the CNS is a rare entity that represents an important differential consideration in the work-up of space occupying lesions of the CNS.

## Background

Granulomas are compact collections of inflammatory cells classically resulting from persistence of a non-degradable product or cell mediated hypersensitivity [1, 2]. While granulomatous involvement of the peripheral nervous system is a well-defined entity in patients with granulomatous disorders such as sarcoidosis, central nervous system granuloma involvement is a rarer entity [3–7].

Intracranial granulomatous space occupying lesions are sparsely reported in the literature and are predominantly caused by infection, retained surgical or foreign objects, or granulomatous disorders. As these lesions are not primarily found in a specific intracranial fossa, the presenting symptoms vary greatly as the inflammation and mass lesion can cause local and distant irritation leading to focal and/or global neurological deficits depending on the location. Moreover, the radiographical features of these lesions have not been well defined and range from diffuse edema to contrast enhancing mass lesions resembling tumors [8]. Here, we present the case of a 77-year-old female who was found to have a necrotizing granulomatous mass found incidentally on imaging which mimicked malignancy.

## Case presentation

**Fig. 1:**
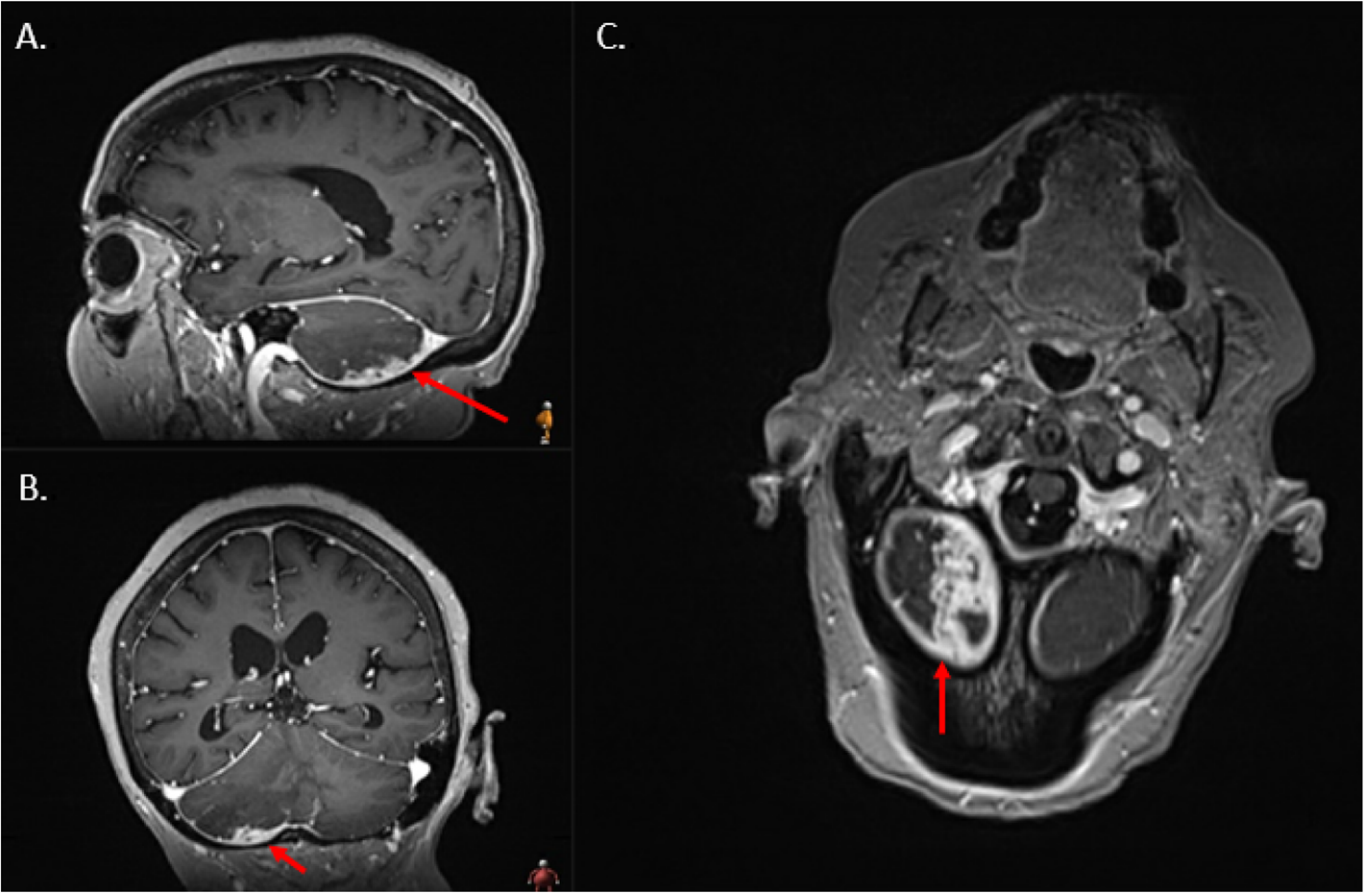
T1-weighted MRI with contrast demonstrates a right dural-based enhancing mass (red arrows) within the posterior fossa along the inferior border of the right cerebellar hemisphere on **a.** sagittal **b.** coronal and **c.** axial reformatted images

**Fig. 2:**
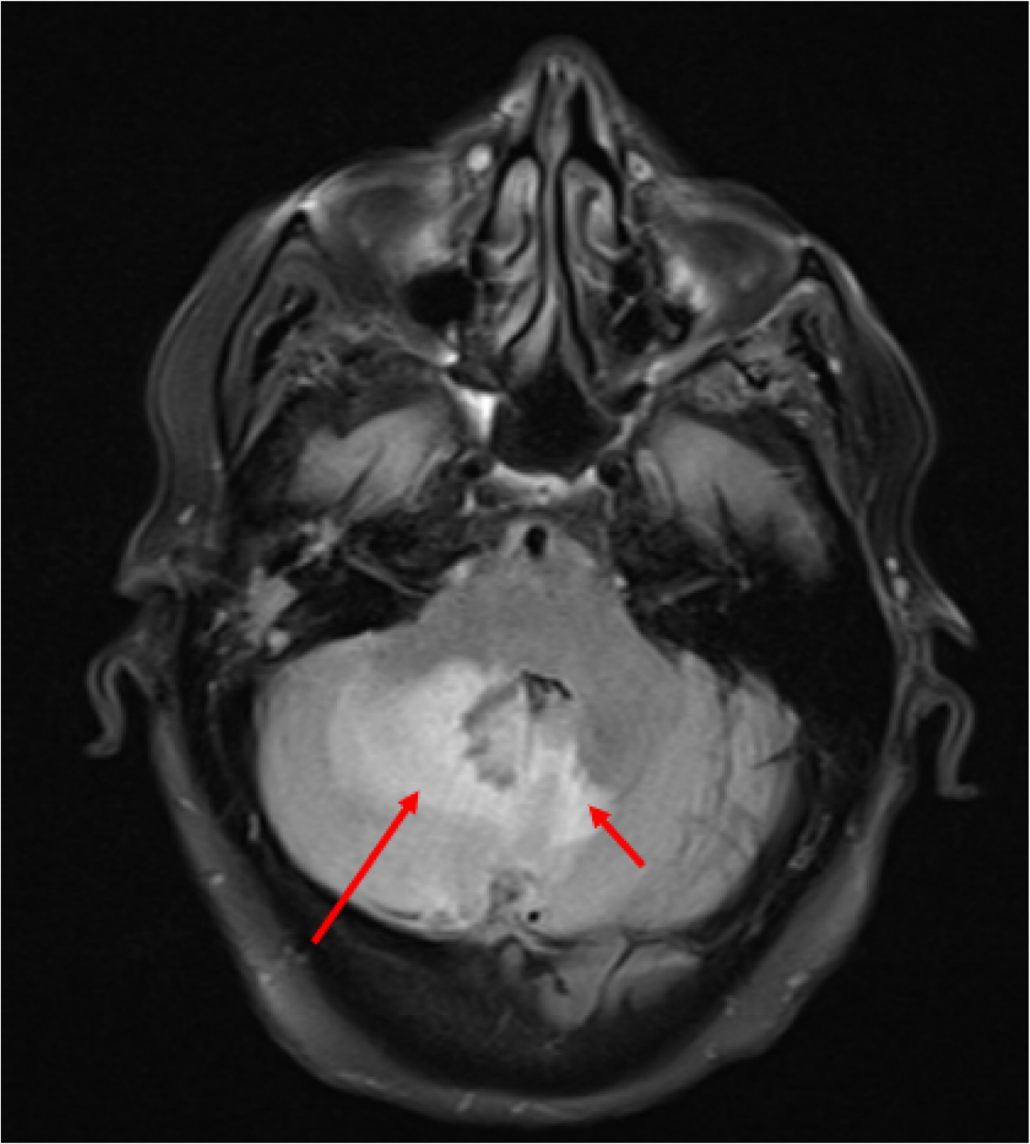
Axial T2 FLAIR sequence MRI at the level of the 4th ventricle within the posterior fossa demonstrating diffuse right cerebellar hyperintensity (long red arrow) and to a lesser extent left cerebellar hemisphere hyperintensity (short red arrow) corresponding to the large amount of vasogenic edema produced by the lesion

A 77-year-old female with a history of chronic kidney disease and type II diabetes mellitus underwent workup for ongoing sinusitis, fatigue, malaise, and 20-pound weight loss. Patient quit smoking 30 years ago and denies any drug or alcohol use. She reports no significant family history including history of malignancy. MRI of the brain performed at an outside hospital to evaluate the extent of sinusitis revealed a posterior fossa lesion with surrounding edema causing compression on the fourth ventricle. The patient was transferred to our institution for neurosurgical evaluation. On presentation the patient was found to have mild cerebellar signs but an otherwise non-focal exam. Patient was surprised to learn of the cerebellar findings considering her lack of significant symptoms. MRI of the brain, including 3DT1, and T2 Flair showed an irregular enhancing lesion along the inferior & posterior surface of the right cerebellar hemisphere suggestive of an infiltrative malignancy (Figs. 1 and 2). Based on these imaging findings, top differentials at the time included a metastatic disease process, an atypical meningioma, or a glioma. Upon retrospective review of this case and imaging findings, an additional differential was hypertrophic pachymeningitis. Contrast CT of the chest, abdomen, and pelvis was then performed and found to be negative for a primary malignancy. H1-MR-spectroscoy was not considered prior to resection. After discussion with the patient and her family, she elected to undergo open biopsy with or without further resection of the lesion. One week after her initial referral, she was taken to the operative theater and underwent a suboccipital craniotomy.

**Fig. 3:**
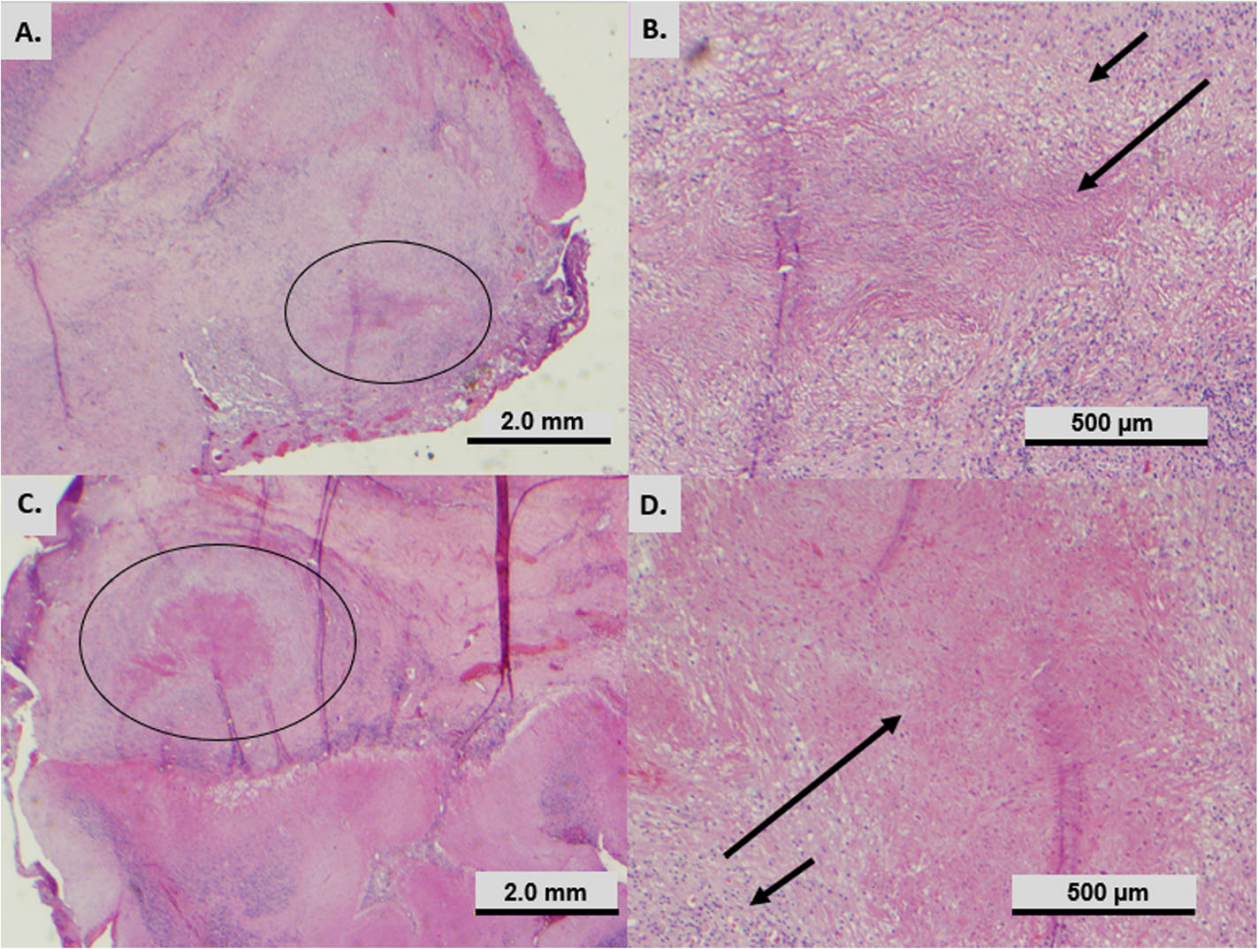
Hematoxylin-and-eosin stained sections of tissue removed from patient. **a** 20x magnification of granuloma (circle) in the cerebellum. **b** 100x magnification showing epithelioid histiocytes (short arrow) and central necrosis (long arrow) of granuloma from image A. **c** 20x magnification of granuloma (circle) in the dura. **d** 100x magnification showing epithelioid histiocytes (long arrow) and central necrosis (short arrow) of granuloma in the dura

An intraoperative frozen section biopsy was taken. Sections showed round or “whorled” structures, suggestive of meningioma. Additional tissue for permanent sections was requested. Permanent sections showed more of the well-circumscribed structures. Without frozen section artifact, the structures could be definitively characterized as necrotizing granulomas, involving both dura and cerebellum. The granulomas comprise central eosinophilic necrosis with surrounding epithelioid histiocytes and lymphocytes (Fig. 3). No vasculitis was seen. Acid fast and Gomori methenamine silver (GMS) special stains were performed; they revealed no acid fast or fungal organisms.

Given the frozen histology, imaging findings, and cerebellar symptoms further resection was completed to the point of gross total resection. cANCA and pANCA studies were sent and were found to be negative. The patient was discharged home on post-operative day 5 with a three-day steroid taper and referral to outpatient physical therapy. She returned for an office follow-up after 6 weeks and was informed that despite extensive work-up to explore the etiology of her granulomatous inflammation, no etiology was found. She had a follow-up MRI at this time which showed a non-enhancing fluid collection extending beyond the titanium mesh cranioplasty, differential to include seroma and pseudomeningocele. A follow up in 2 weeks was planned to monitor this fluid collection. At this appointment, it was found that the fluid collection under the incision was significantly decreased in size and continuing to resolve. Patient stated she was overall doing well. The current plan is to follow up in 1 year with an MRI. Patient stated she was happy with her recovery progress and plan of care moving forward.

## Discussion and conclusions

Granulomas are compact collections of inflammatory mononuclear or multinuclear cells classically resulting from persistence of a non-degradable product or cell mediated hypersensitivity [1, 2]. Granulomas can be confluent or discrete with varying degrees of necrosis and calcification [2]. The main categories of granulomatous disorders are infections, vasculitis, immunological upsets, leucocyte oxidase defects, hypersensitivity, chemicals, and neoplasia [2]. In light of this broad differential, clinicopathological correlation is required to form a correct diagnosis and treatment plan.

The presentation of intracranial granulomas, regardless of etiology, depends on location in the brain. Symptoms reported as a result of intracranial granulomatous masses include seizures and extremity weakness (right parafalcine region, parietal lobe), and visual disturbances (occipital lobe) [9–11]. It is much more common for granulomatous involvement in the head and neck to be in the sinonasal cavity (presents as sinusitis and polyposis), the orbit (presents as scleritis, lacrimal gland enlargement, and vision loss), temporal bone and skull base (presents with cranial nerve loss, otitis media, and hearing loss), and small vessels (which can result in vessel occlusion) [12]. Our patient had the necrotizing granulomatous mass located in her cerebellum. She presented with mild cerebellar findings only.

Autoimmune granulomatous disorders with known head and neck manifestations include Behcet disease, Churg- Strauss syndrome, and granulomatous polyangiitis (GPA, previously called Wegener granulomatosis) [12]. All three of these disease processes are types of vasculitis, with several key differences. Behcet disease typically causes mucosal ulcerations, uveitis, and sensorineural hearing loss in the head and neck [12]. Churg-Strauss syndrome presents with allergic rhinitis and nasal polyposis. GPA is a multisystem necrotizing small vessel vasculitis with a classic triad of nose/throat, lung, and renal involvement [12, 13]. GPA is commonly associated with antineutrophil cytoplasmic antibodies; however, 40% of patients with no renal involvement are ANCA negative [13]. Our patient has a history of sinusitis and interstitial lung disease but no lung lesions. The patient has no known renal involvement (no hypertension, no urinary symptoms, normal urinalysis, and no masses found on imaging although a renal biopsy has not been performed) and is ANCA negative; therefore, the diagnosis of GPA remains a possibility. Azuma et al. and Nicolosi et al. have both previously described a patient with GPA presenting with an intracranial necrotizing granulomatous lesion [9, 11]. It is important to note, however, that only 2–6% of GPA cases [11] have central nervous system (CNS) involvement.

Other possible causes of intracranial granulomas include sarcoidosis, foreign bodies, IgG4-related hypertrophic pachymeningitis, and infectious etiologies. Neurosarcoidosis is present in 5% of patients with systemic sarcoid [14]. There have been 100 cases of intracranial granulomas as of December 2018 due to foreign bodies remaining after cranial surgeries [15]. Tuberculomas, a common infectious etiology, represent 10 to 30% of all intracranial space occupying lesions in patients in tuberculosis endemic zones [16]. Our patient has no history of sarcoidosis, and no prior surgery that could have introduced a foreign object. Histology was not suggestive of IgG4-related hypertrophic pachymeningitis, as neither “storiform” fibrosis, nor obliterative phlebitis, nor abundant plasma cells were seen. There was also no evidence of infection in this case, and as of 2015 there were only 11 reported cases of tuberculomas in the cerebellum (the location of this patient’s lesion) [16]. Moreover, special stains for microorganisms were negative.

In light of the exclusion of other possibilities described above, a spontaneous necrotizing granulomatous mass is the best explanation in this case. There has only been one case reported of a spontaneous intracranial granuloma that mimicked a brain tumor [10]. The patient in that case was a female with a history of scleroderma, and was treated successfully with steroids [10].

This is the second reported case of likely spontaneous necrotizing intracranial granuloma, although ANCA negative GPA cannot be completely excluded. Though rare, spontaneous necrotizing granuloma is an important consideration in the assessment of intracranial mass lesions when other more common etiologies have been excluded.

## Data Availability

Data sharing is not applicable to this article as no data sets were generated or analyzed during the current study.

## Abbreviations

ADC: Apparent diffusion coefficient
ANCA: Antineutrophil cytoplasmic antibodies
c-ANCA: Cytoplasmic antineutrophil cytoplasmic antibodies
CNS: Central nervous system
CT: Computerized tomography
GMS: Gomori methenamine silver
GPA: Granulomatous polyangiitis
MRI: Magnetic resonance imaging
p-ANCA: Perinuclear antineutrophil cytoplasmic antibodies
